# A descriptive study of medical educators' views of problem-based learning

**DOI:** 10.1186/1472-6920-9-66

**Published:** 2009-11-04

**Authors:** Mohsen Tavakol, Reg Dennick, Sina Tavakol

**Affiliations:** 1 Medical Education Unit, The University of Nottingham, Queen's Medical Centre, UK; 2 School of Biomedical Sciences, The University of Nottingham, Queen's Medical Centre, UK

## Abstract

**Background:**

There is a growing amount of literature on the benefits and drawbacks of Problem-Based Learning (PBL) compared to conventional curricula. However, it seems that PBL research studies do not provide information rigorously and formally that can contribute to making evidence-based medical education decisions. The authors performed an investigation aimed at medical education scholars around the question, "What are the views of medical educators concerning the PBL approach?"

**Methods:**

After framing the question, the method of data collection relied on asking medical educators to report their views on PBL. Two methods were used for collecting data: the questionnaire survey and an online discussion forum.

**Results:**

The descriptive analysis of the study showed that many participants value the PBL approach in the practice and training of doctors. However, some participants hold contrasting views upon the importance of the PBL approach in basic medical education. For example, more than a third of participants (38.5%) had a neutral stance on PBL as a student-oriented educational approach. The same proportion of participants also had a neutral view of the efficiency of traditional learning compared to a PBL tutorial. The open-ended question explored the importance of faculty development in PBL. A few participants had negative perceptions of the epistemological assumptions of PBL. Two themes emerged from the analysis of the forum repliers: the importance of the faculty role and self-managed education.

**Conclusion:**

Whilst many participants valued the importance of the PBL approach in the practice and training of doctors and agreed with most of the conventional descriptions of PBL, some participants held contrasting views on the importance of the PBL approach in undergraduate medical education. However there was a strong view concerning the importance of facilitator training. More research is needed to understand the process of PBL better.

## Background

PBL is possibly one of the most innovative themes in medical education; it has raised extreme debate and still continues to generate passionate discussions. There is a growing amount of literature on the benefits and drawbacks of PBL compared to conventional curricula. The experimental studies reported in the three reviews published in 1993 [1-3] showed that there is a dearth of good quality studies and evidence available regarding the hypothesis that PBL produces learners different to or superior to those derived from traditional methods [4]. This has led supporters and detractors to continue to investigate further the epistemological and ontological issues arising from the processes and outcomes of PBL. However, it has been asserted that the quality of medical education research is poor, repetitive, not informed by theory, methodologically weak and does not pay attention to validity threats in quasi-experimental designs [5,6]. A critical reading of studies on the methods and findings of PBL showed that they had not provided an evidence-base indicating the educational superiority of PBL despite the fact that such studies underpinned the effectiveness of PBL on attitudes, perceptions, self-rating and opinions [7]. It has also been argued that all forms of research involving subjectivity such as ethnography, grounded theory and phenomenology have been "unscientific" due to a lack of explicability, repeatability and replicability [8]. Therefore, qualitative studies, which explored the experiences and perceptions of students and tutors in programs that incorporated student-centred problem-based pedagogy, may not provide the best available evidence for the effectiveness of PBL curricula. Similarly, quantitative studies which compared the PBL approach with conventional teaching, might not illustrate the potential impact that it can have, if statistical effect size measures are not reported [9].

With respect to learning theories, PBL arose from the personal experiences and beliefs of a few medical educators [10] and it was arguably non-theoretical in its development. However, as PBL has evolved, some learning theories were claimed to support PBL [11]. In medical education, PBL has its roots in constructivist theories of learning [12]. However, Colliver has asserted that constructivism is not a theory of learning. "It provides a fleeting insight into the learning process, but it is not a theory of learning. It confuses epistemology and learning, and it would seem to offer little of value to medical education" [13]. Furthermore, when appraising some PBL quantitative papers, we noticed that the studies were not based on any learning theory or were not testing predictions from a learning theory. If a study tests a prediction or hypothesis based on a theory and the findings are consistent with the theory, then the findings are considered to support that theory [14]. Learning theory has not been used to design quantitative PBL studies and data from studies has not been used to support theory. Perhaps corruptions of quantitative inquiry approaches in recent years place the credibility of PBL at stake, and it may be argued that the findings generated are trivial or obvious.

Taken together, these ideas seem to indicate that PBL research studies do not provide information rigorously and formally that contribute to making evidence-based medical education decisions. Perhaps for this reason medical education scholars are still uncertain whether the PBL approach creates better physicians compared with traditional learning, or whether the PBL approach is superior to didactic basic and clinical teaching. Is "the glass half-full"? [15] or just "half empty?" [16]. While the benefits of curriculum reform are strongly cited, especially the increased use of PBL, there is a dearth of research assessing the effects of various curricula including PBL on preclinical and clinical measures of student performance. The exception to this is the longitudinal study on the impact of various curricula (including PBL) on student learning once they begin clinical practice. The authors concluded that changing curricula in medical education reform is not likely to have an impact on improvements in student achievement [17]. We do agree with Wood who stated that "performing outcomes based research in education is difficult because of the large range of confounding factors" [18]. Contrary to the conclusion of Wood, it seems that, for PBL, we do need to continue "arguing about the process and examine outcomes". This may bolster the promise of replication studies, which are necessary for the formation of a body of best evidence-based medical education practice, particularly for PBL.

We felt it important, therefore, to conduct a study, which is grounded in the benefits and drawbacks of current PBL research findings. We asked ourselves: What are the views of medical educators concerning the PBL approach? This study provides a new picture that may add to our overall understanding of PBL.

## Methods

The study started in March 2006 in the UK, with a planned recruitment period of 18 months. The method of data collection relied on asking medical educators to report their views in a survey. Ethical approval was not sought as this was an opportunistic sample from volunteers at a one-day conference and web-based survey and by opting to reply to the questionnaire [see additional file 1], the participants automatically agreed to take part in this study, and consequently a consent form was not presented to them. The survey was an anonymous study. Two methods were used for collecting data. Firstly, questionnaires were distributed to a convenience sample of 65 medical educators, who participated in the 3^rd ^UK conference on Graduate Entry Medicine (GEM), 14^th ^July 2006. The number of completed, useable questionnaires was 33, giving a response rate of 51%. This low response rate led us to collect questionnaire data through the Internet in order to increase the sample size. For this, we embedded the same questionnaire in a web application that was only accessible through a confidential hyperlink. After a list of potential respondents was created (n = 27), an email including the hyperlink was sent out to the members of that list inviting them to participate in this study. The use of follow up reminders was ineffective in achieving higher response rate for the web-based survey. Six medical educators filled in the web-based survey. Table 1 shows the characteristics of participants.

**Table 1:** Demographic characteristics of study participants (n = 39)

**Characteristic**	**No (%)**
**Gender**	
Female	24(61.5)
Male	14 (35.9)
Missing	1 (2.6)
Total	39(100)

**Age**	
21-30	2(5.1)
31-40	7(18.0)
41-50	12(30.8)
51-60	8(20.5)
61-70	2(5.1)
Missing	8(20.5)
Total	39(100)

**Student admission**	
GEM	14(35.9)
School-leavers	15(38.5)
Both	9(23.0)
Missing	1(2.6)
Total	39(100)

**Having health professional qualification**	
Yes	22 (56.4)
No	17 (43.6)
Total	39(100)

**Having experience of conventional course**	
Yes	37(94.9)
No	2 (5.1)
Total	39(100)

The second method for collecting data was a discussion forum entitled "What have we learned from the PBL approach?" An email was forwarded to the members of the Evidence Based Medical Education (EBME) collaboration in order to ascertain their view on PBL. We asked medical educators who have experienced PBL to discuss their views on the PBL approach. Six members commented regarding the above question in a forum discussion. Therefore, in this study the purposive sample consisted of 39 medical education scholars and 6 forum repliers, with firsthand experience of PBL.

The design of the questionnaire was based on a thorough review of the literature relating to PBL studies. The PBL scale consists of 17 items about the conditions that hinder and support PBL. To reduce the bias of the questionnaire, some items were written negatively, so that not all questions reflected positive views towards PBL. Each item was accompanied by a 5-point Likert scale, ranging from 1 (strongly disagree) to 5 (strongly agree). An open-ended question was provided to find out the medical educator's experience concerning the PBL approach. Medical educators also provided demographic information, which included items on their age, gender, and experience. A brief instruction for the completion of the study instrument was provided to ensure that it could be self-administered.

Prior to conducting the survey, the content validity of the instrument was established by subjecting it to review by two PBL experts. The experts were selected based on their deep experiences of PBL and their knowledge of the PBL process in their own school. We asked them to criticise the statements if they did not make sense or cover the purpose of the study. We took their comments on the questionnaire design into consideration, and we made appropriate modifications to clarifying meaning. We then tested the questionnaire for reliability with data from a group of individual participants (n = 20). The reliability of the tool was determined by computation of Chronbach's alpha using SPSS, which gave a value of 0.68, indicating an acceptable degree of internal consistency.

Because this study was primarily descriptive, descriptive information was presented for numerical data analysis. Words or sentences provided by participants in the open-ended questions have been reported in a table. The forum replies were also read and re-read in order to identify emerging themes as headings under which we can categorise most of the data.

## Results

The results of this study can best be treated under three headings: the PBL scale, open-ended question, and forum repliers.

### The PBL scale

A sample of 39 medical educators from an accessible population was recruited (Table 1). The mean number of years work experience with facilitating was 7 years (SD 6.3, minimum 1 year, and maximum 30 years). Participants were asked to rate the extent to which they perceived each of 17 items. Responses to agree and strongly agree were combined as "agree" and to disagree and strongly disagree were combined as "disagree". Most (69.2%) of respondents agreed that there is a difference between a PBL course and a conventional course. When asked to report whether they experienced PBL as a student- centred approach, more than a third of the respondents (36.2%) agreed. In response to the item, 'the facilitator needs to be expert in the subject matter of the case', the majority of respondents (61.6%) disagreed. More than a half of the respondents (51.3%) disagreed that 'Learning from a large group lecture is a more efficient way of learning than a PBL tutorial. Some respondents (35.9%) felt 'neutral' about increasing the number of doctors in the UK using graduate entry PBL. Most educators (62%) disagreed that the facilitator is not redundant in a PBL tutorial meeting. More than one fourth of respondents (25.7%) agreed that students are forced to participate in PBL by the facilitator. Few respondents (15.4%) had a neutral view on this. When asked to report if a lecture-based environment makes for better job satisfaction compared with a PBL course, more than a half of the respondents (51.3%) disagreed. The majority of participants (51.2%) disagreed that the students on a PBL course spend too much time elaborating their knowledge in comparison with a conventional course. As Table 2 shows, many participants valued the importance of the PBL approach in the practice and training of doctors. However, some participants held contrasting views upon the importance of the PBL approach in undergraduate medical education. For example, the scores showed that most participants had a neutral view of the efficiency of lecture-based learning compared to a PBL tutorial.

**Table 2:** Responses of medical educators to PBL views statements

**Item**	**SD**		**D**		**N**		**A**		**SA**		M*	
	**N**	**%**	**N**	**%**	**N**	**%**	**N**	**%**	**N**	**%**	**N**	**%**

1. There is a significant difference between a PBL course and a conventional course	3	7.7	2	5.1	5	12.8	14	35.9	13	33.3	2	5.1

2. PBL is a student-centred approach	2	5.1	3	7.7	15	38.5	12	30.8	6	15.4	1	2.6

3. The facilitator needs to be expert in the subject matter of the case	12	30.8	12	30.8	8	20.5	4	10.3	3	7.7	0	0

4. Learning from a large group lecture is a more efficient way of learning than a PBL tutorial	14	35.9	6	15.4	15	38.5	3	7.7	1	2.6	0	0

5. Knowledge is better acquired in a lecture based course rather than a PBL based course	8	20.5	8	20.5	15	38.5	7	17.9	1	2.6	0	0

6. PBL makes the transition easier from school to the medical environment	7	17.9	8	20.15	15	38.5	4	10.3	3	7.7	2	5.1

7. PBL is compatible with the way that I understand my specialty or subject area	2	5.1	1	2.6	10	25.6	16	41.0	10	25.6	0	0

8. Graduate entry PBL is a more effective way of increasing the number of doctors in the UK	6	15.4	8	20.5	14	35.9	7	17.9	2	5.1	2	5.1

9. Graduate entry PBL will create doctors who have come from a greater variety of educational backgrounds	6	15.4	11	28.2	9	23.1	10	25.6	3	7.7	0	0

10. The facilitator is redundant in a PBL tutorial meeting because students can manage their own "case scenario"	11	28.2	21	53.8	3	7.7	2	5.1	2	5.1	0	0

11. Students are forced to participate in PBL by the facilitator	10	25.6	9	23.1	6	15.4	9	23.1	1	2.6	4	10.3

12. Graduate entry PBL will create better doctors because they have greater maturity and life experiences	4	10.3	5	12.8	16	41.0	10	25.6	2	5.1	2	5.1

13. Colleagues, who teach in a lecture based environment, have better job satisfaction than those who teach on a PBL course	14	35.9	6	15.4	8	20.5	7	17.9	1	2.6	3	7.7

14. Students on a PBL course invest too much time elaborating their knowledge in comparison with a conventional course	10	25.6	10	25.6	11	28.2	4	10.3	2	5.1	2	5.1

15. A lot of effort is needed to implement a PBL course	0.0	0.0	1	2.6	0.0	0.0	20	51.3	17	43.6	1	2.6

16. People should have considered more educational evidence before implementing PBL courses	5	12.8	8	20.5	12	30.8	5	12.8	8	20.5	1	2.6

17. PBL students have more confidence in questioning and interacting when they are in taught classes	1	2.6	4	10.3	15	38.5	12	30.8	7	17.9	0	0

*Strongly disagree (SD), Disagree (D), Neutral (N), Agree (A), Strongly agree (SA), Missing system (M)

### The open-ended question

Respondents were asked in an open ended question for their opinions on lessons they have learned or experienced during PBL tutorials. Of note was the low response to this question. For this reason, we analysed words and terms provided by the participants (Table 3). It is apparent from this table that the participants had concerns about issues relating to facilitators (items 3, 5, 6). The findings also indicated the importance that participants placed on student learning in PBL. One participant had concerns about the use of the PBL approach for Graduate Entry Medicine.

**Table 3:** Words or phrases that participants used to describe the lessons learned from the PBL approach by 8 participants

1.	PBL is still unclear in GEM
2.	Need to ensure students are fully aware before studying the course regarding education, using PBL and its implementations, especially in regard to their learning style. Need to give students guidance on the boundaries of knowledge they need to have to allow PBL to operate efficiently and then to feel secure.
3.	Make sure the facilitators are well trained and follow the proper methods so all students get same experience.
4.	Have seen shy students coming out of their shell as they progress. Have seen them becoming more confident and develop social skills.
5.	Revisit group rules- when things aren't going well. Train the facilitator to confront blocks in groups. Good facilitators are more important than subject experts.
6.	Need to be well organised. Facilitator should have greater ability and training to be effective. Otherwise a lot of time will be wasted.
7.	Important to have time up-front with the group to engage in introductions and ground rule development before starting work.

### The forum repliers

Two general themes emerged from the forum repliers concerning medical educators' experiences of the PBL approach. They are: faculty role and self-managed education. We will now look at each of these in turn.

#### Faculty role

Participants in the forum had different views with respect to the PBL approach. One participant, who had graduated in medicine and experienced PBL, reflected that the PBL approach was useful in teaching 3^rd ^year medical students who are just entering their clinical training. This is because students integrate basic science with clinical application. Although one of the principal ideas behind PBL is that students aim their learning at the areas in which their knowledge is more deficient, one participant asserted that students sometimes "*do not know what they don't know*". This finding may show that students are 'unconsciously incompetent', on the first stage of the conscious-competence framework in PBL. The participant described the facilitator role as crucial to effective learning in the PBL tutorial. He continued that students who had a process expert in discussion failed to catch key concepts and key pieces of information in their literature searching, or key insights in terms of understanding the questions they are addressing. The situation described below exemplifies this behaviour:

"...on the one hand clear objectives and faculty development are necessary so students are properly advised through the PBL exercise, such that they take ownership of their own learning and true self-directed learning can happen. Especially early in medical training, students cannot know what they need to learn in order to solve the problem. On the other hand, affirmation from a tutor that students are on the right track can very easily turn into direction from the tutor, and that can turn into teaching by the tutor."

A consultant stressed the importance of the faculty role in the PBL approach and strategies for successful facilitation. He found that the main barrier to implementation of PBL is the lack of preparation of faculty members to facilitate self-directed learning.

#### Self-managed education

In terms of the student-centred nature of the PBL approach, with its emphasis on self-directed learning, one respondent stated that the learning objectives of a PBL course do not provide the opportunity to encourage students to take greater ownership of their work, and hence greater responsibility for their learning. A participant replied:

"If clear learning objectives are prepared (not a list of subjects or objectives that use ambiguous words such as 'to understand'), and a series of concepts or principles are identified (those that the faculty think can be missed by the students), then the students can become truly a self-directed learner exerting a high degree of autonomy."

Another participant reflected on this situation:

"For me, the key question is, to what degree we believe in self-directed learning and convey to the students the message that they can take responsibility for their learning without our intervention?"

These perceptions indicate that the self-directed nature of PBL is still challenging. This may show that the participants interpreted self-directed learning as surface oriented self teaching. As such, this may indicate the students do not have control over all elements of the PBL process. Students, for example, have no control over the scenario, although the nature of self-directed learning of PBL is acknowledged.

## Discussion

This study has methodological limitations that must be taken into consideration when interpreting the findings. One cannot over-emphasise the limitations of self-report as this may limit the validity of findings. Respondents for various reasons may under, or overestimate their practice. A methodological problem frequently associated with the use of self-reports in questionnaires, which may have been evident in the present study, is the inability to determine the extent to which responses accurately reflect the respondents' experiences and expectations of their PBL tutorial sessions. This warrants further research to examine the actual PBL process. It is also possible that medical educators in this study were not representative of PBL educators.

The response rate was low, despite our efforts to maximise it and this means that the findings should be interpreted with caution. Reasons for non-response are not known. Non-respondents to the survey may also be less interested or involved in PBL, and therefore the reported extent of the PBL approach in this study may be higher than in reality.

Regarding forum repliers, this was a convenience sample consisting of only 6 medical educators. The online forum discussions were convenient and provided a transcribed record. Drawbacks to participation in online discussions may be the same as for online education in general, that is, the inability to capture the richness and depth of meaning without visual and verbal clues.

To overcome these methodological limitations we suggest, therefore, randomised experiments which focus on the performance of PBL graduates and non-PBL graduates in the clinical workplace. This may optimise the accuracy of inferences about the PBL approach. Clearly, an important task facing researchers is the identification and control of those factors that may give rise to alternative explanations for the effects of PBL compared to non-PBL methods. Factors such as the educational background of the students, methods of student selection and the learning culture of the institution are all potentially important. In addition perhaps more emphasis should be placed on researching the comparative learning processes that PBL and non-PBL students engage in. For example PBL students engage in considerably more verbal discourse, questioning and reasoning episodes than traditional students. Perhaps this develops additional cognitive and interpersonal skills not necessarily acquired to the same extent by more didactic and teacher-centred learning methods.

The descriptive analysis of this study showed that many participants valued the PBL approach in the practice and training of doctors. However, some medical education scholars held contrasting views upon on the importance of the PBL approach in undergraduate medical education. Among the medical educators surveyed, 38.5% had a neutral experience of PBL as a student-oriented educational approach. This finding is not consistent with the common characteristic of the PBL approach, indicating its student-centred nature [19]. Although 46.2% of participants valued PBL as a student-oriented approach, the question that comes to mind is why do a group of medical educators feel so uncertain about it? Further research should examine this. What is surprising is that more than 61% of medical educators disagreed that the facilitator needs to be an expert in the subject matter of the case despite the fact that the majority of participants had a medical health professional qualification. The issue of content knowledge compared to process expertise is still challenging. Some evidence shows differences in favour of content experts when compared with process expertise [20]. For example, Eagle *et al*. concluded that twice as many learning issues were identified by groups led by content experts [21]. Consistent with the results of these studies, Schmidt *et al *concluded that students guided by subject experts spent more time on self-directed learning and achieved somewhat better scores on high stakes tests than students guided by non-expert facilitators [22]. However, a study by Silver and Wilkerson indicated that content expertise resulted in more tutor-directed discussion in a PBL course [23]. Taken together, these studies may suggest that both subject and process expertise are required by facilitators.

The results of this study indicate that the participants had a neutral view of the efficiency of traditional learning compared to a PBL tutorial. As such, participants had a neutral view of the claim that knowledge is better acquired in PBL-based course rather than a lecture-based one. These findings add to most previous research studies by demonstrating that there is no difference between the knowledge that PBL students and non-PBL students acquire about medical sciences [24]. Although studies show that group learning in PBL may have positive effects, much more empirical evidence is needed to obtain deeper insight into the productive group learning of a PBL tutorial [25]. One may argue that the process of PBL needs to be rigorously investigated in order to offer reasons for believing that it is designed to help student construct an extensive knowledge base and to become doctors dedicated to lifelong-learning. It is therefore important to further explore the nature of the learning acquired from PBL courses compared to traditional instruction courses.

With respect to graduate entry PBL, this study did not show that the policy of admitting graduates versus school-leavers to medical programmes was perceived as effective in creating better doctors. Interestingly, no previous PBL studies have explored differences between graduate entry PBL and school leaver programmes, although this study revealed that graduate entry PBL is not perceived as a more effective way of increasing the number of doctors in the UK by the majority of responders. In addition, this study revealed that there was a majority perception that graduate entry PBL will produce doctors who have come from a greater variety of educational backgrounds. However, will graduate entry PBL create *better *doctors compared to school leaver programmes? Sophisticated methodological approaches are required to answer this question.

The descriptions of medical educators about the PBL approach focused on the process of PBL, the characteristics of a good PBL facilitator and the advantages and disadvantages of PBL. It has been well documented that the facilitator role is central to PBL. The adoption of the role requires an understanding of epistemological and ontological issues about teaching and learning in medicine. In the epistemological sense PBL students are novices and the knowledge facilitator should assist them in restructuring new knowledge based on their prior declarative and procedural knowledge. In the ontological sense perceiving a new reality by students is important and the role of the facilitator is to assist students to explore reality in different ways. As the importance of faculty development in PBL was valued by participants in the forum discussion this may suggest more facilitator development workshops to help achieve competence as skilled facilitators of the PBL process. Such workshops may uncover conflicting roles of tutors in the steps of the PBL process. As Irby indicated, identifying and practicing these roles (mediator, challenger, negotiator, director, evaluator and listener) is a key skill of effective facilitation [26].

In addition to this, one medical educator had a negative approach about PBL, and reflected: "*PBL is still unclear in GEM*". It seems that some medical educators have negative perceptions of the ontological assumptions of PBL. For instance, a qualitative study was conducted to explore how a cohort of tutors made sense of PBL. In this study, one participant stated: "*absolutely not, no views not really changed at all. I'm still not convinced that PBL, despite the fact that [I will tutor again] is the proper way of teaching" *[27]. Altogether these findings concerning academic achievement are slightly in favour of non-PBL programmes.

When asked about their experience in a PBL tutorial course, medical educators indicated they had few negative feelings with respect to facilitating self-directed learning and student learning. There are several possible reasons for this. Firstly, in the beginning of the course, it seems that the students find adopting a self-directed problem-based approach to learning difficult as they "do not know what they do not know". This may be attributed to the fact that students may have a restricted personal knowledge of the complexity of the "case". Secondly, students may not have clear objectives for the behaviour that they have to achieve, particularly in clinical settings, as mentioned by one participant. Thirdly, learning styles, both deep, surface and 'strategic', are determined at secondary school, and it is also difficult to influence learning styles even with a PBL curriculum [28,29].

In this study, a few participants suggested combinations of pedagogical strategies, where several PBL courses are offered along with courses presented in a more traditional way. There is no evidence that indicates how a hybrid curriculum can make students better doctors compared to other approaches. However, a recent study concluded that changing curricula in medical education reform is not likely to have an impact in improvement in student achievement [17]. The authors suggested that further work ought to focus on student characteristics and teacher characteristics such as teaching competency.

## Conclusion

Whilst many participants valued the importance of the PBL approach in the practice and training of doctors and agreed with most of the conventional descriptions of PBL, some participants held contrasting views upon the importance of the PBL approach in undergraduate medical education. For example, most participants had a neutral view of the efficiency of lecture-based learning compared to a PBL tutorial. However there was a strong view concerning the importance of facilitator training. We need to understand the process of PBL better.

## Competing interests

The authors declare that they have no competing interests.

## Authors' contributions

All authors contributed to the conception and design of the study. MT, RD and ST contributed to the acquisition, analysis and interpretation of data. MT and RD contributed to drafting of the paper. All authors contributed to the critical revision of the paper and approved the final paper for publication.

## Pre-publication history

The pre-publication history for this paper can be accessed here:



## Supplementary Material

Additional file 1 The study instrument. Click here for file
