# Supplementary material for: A descriptive study of medical educators' views of problem-based learning
Source: BMC Med Educ. 2009 Nov 4;9:66. doi: 10.1186/1472-6920-9-66 (PMC2775736; doi:10.1186/1472-6920-9-66)
Supplement: Additional file 1 — The study instrument. [file 1472-6920-9-66-S1.doc]

The following questionnaire has been produced as part of a research project regarding the problem-based learning approach. All responses are confidential. Please answer all questions as honestly as possible. Thank you for your cooperation.

Years

**Age: Gender: Male Female**

**Your students are: Graduates School-leavers Both**

Years

**Years of experience with facilitating:**

**Do you have a non-medical health professional qualification? Yes No**

**Have you experienced of “conventional medical courses”? Yes No**

***Please rate the following statements on a scale from 1-5, where 1= disagree and 5 =agree strongly***

1. There is a significant difference between a PBL course and a conventional course.

Disagree1 2 3 4 5 Agree strongly

1. I recommend PBL as a method of learning medicine.

Disagree1 2 3 4 5 Agree Strongly

3. PBL is a student-centered approach.

Disagree 1 2 3 4 5 Agree Strongly

1. The facilitator needs to be expert in the subject matter of the case.

Disagree 1 2 3 4 5 Agree Strongly

1. Learning from a large group lecture is a more efficient way of learning than a PBL tutorial.

Disagree 1 2 3 4 5 Agree Strongly

1. Knowledge is better acquired in a lecture based course rather than a PBL based course.

Disagree 1 2 3 4 5 Agree Strongly

1. PBL makes the transition easier from school to the medical environment.

Disagree 1 2 3 4 5 Agree Strongly

1. PBL is compatible with the way that I understand my specialty or subject area.

Disagree1 2 3 4 5 Agree Strongly

1. Graduate entry PBL is a more effective way of increasing the number of doctors in the UK.

Disagree 1 2 3 4 5 Agree Strongly

1. Graduate entry PBL will create doctors who have come from a greater variety of educational backgrounds

Disagree 1 2 3 4 5 Agree Strongly

10. Graduate entry PBL will create better doctors because they have greater maturity and life experiences

Disagree 1 2 3 4 5 Agree Strongly

11. The facilitator is redundant in a PBL tutorial meeting because students can manage their own “case scenario”.

Disagree1 2 3 4 5 Agree Strongly

12. Students are forced to participate in PBL by the facilitator.

Disagree 1 2 3 4 5 Agree Strongly

13. Colleagues, who teach in a lecture based environment, have better job satisfaction than those who teach on a PBL course.

Disagree 1 2 3 4 5 Agree Strongly

14. Students on a PBL course invest too much time elaborating their knowledge in comparison with a conventional course.

Disagree 1 2 3 4 5 Agree Strongly

15. A lot of effort is needed to implement a PBL course.

Disagree 1 2 3 4 5 Agree Strongly

16. People should have considered more educational evidence before implementing PBL courses.

Disagree 1 2 3 4 5 Agree Strongly

17. PBL students have more confidence in questioning and interacting when they are in taught classes.

Disagree 1 2 3 4 5 Agree Strongly

18. What lessons have you learned or experienced during PBL tutorials, that you have facilitated, which could be helpful for other medical schools to improve the way they implement and organize a new PBL course. Please indicate them briefly.

………………………………………………………………………………………………………………………………………………………………………………………………………………………………………………………………………
